# Bone-Loading Physical Activity and Alcohol Intake but not BMI Affect Areal Bone Mineral Density in Young College-Aged Korean Women: A Cross-Sectional Study

**DOI:** 10.3390/ijerph16245063

**Published:** 2019-12-12

**Authors:** Dong Jun Sung, Harshvardhan Singh, Seung-Bum Oh, SoJung Kim

**Affiliations:** 1 Division of Sport and Health Studies, College of Biomedical and Health Science, Konkuk University, Chungju-si 27478, Korea; sls98@kku.ac.kr (D.J.S.); osb0509@kku.ac.kr (S.-B.O.); 2 Sports Convergence Institute, Konkuk University, Chungju-si 27478, Korea; 3 Department of Physical Therapy, University of Alabama at Birmingham, 1716 9th Avenue South, Birmingham, AL 35294, USA; hsingh@uab.edu; 4 Department of Physical Therapy and Kinesiology, University of Massachusetts Lowell, 3 Solomont Way, Lowell, MA 01854, USA

**Keywords:** bone mineral density, bone-specific physical activity, body mass index, young women

## Abstract

The purpose of this cross-sectional study was to determine the differences in areal bone mineral density (aBMD) based on alcohol consumption behaviors, bone-loading history as assessed by a bone-specific physical activity questionnaire (BPAQ), and the body mass index (BMI). College-aged female students (*N* = 112) were recruited from the universities in Seoul and Gyeonggi province, South Korea. The aBMD of the lumbar spine and non-dominant side of the proximal femur (total hip, TH; femoral neck, FN; femoral trochanter, FT) were measured using dual energy X-ray absorptiometry (DXA). Alcohol consumption was determined by the frequency and amount of alcohol intake during the past 12 months using a self-reported questionnaire. The X-scan plus II was used to measure height (cm), body mass (kg), fat-free mass (FFM, kg), and % body fat. Drinking two or more times alcohol per week was associated with greater aBMD of the TH (*p* = 0.04–0.002) and FN (*p* = 0.043) compared to a lower frequency of alcohol consumption and 2–4 times per month, respectively. Based on the drinking amount per occasion, there were no significant group differences (*p* > 0.05) in aBMD at any of the sites. The highest group of total BPAQ had greater aBMD of the TH, FN, and FT versus the lowest (*p* = 0.023–0.009) and mid of total BPAQ groups (*p* = 0.004–0.009). Additionally, the highest group had greater aBMD of the lumbar spine compared to the mid group (*p* = 0.001). No significant group differences in aBMD at any of the sites were noted based on the BMI (*p* > 0.05). Young college-aged women with greater bone-loading physical activity showed greater aBMD at the TH, FN, FT, and lumbar spine, while a moderate alcohol intake was associated with greater aBMD of the TH and FN. These findings have clinical implications for young women who may not participate in high-impact physical activity and are binge drinkers.

## 1. Introduction

Skeletal health is critically important for one’s overall health and can improve the quality of life for both young and aging populations [1]. By the end of the third decade of life, bones reach their maximum peak bone mass (PBM), which is vital for future bone strength [2]. Among factors affecting PBM, bone-strengthening activities (e.g., jumping, dynamic movements, and high-intensity resistance training) [2,3,4,5], a proper amount of calcium [6], and vitamin D intake [1] have been shown to be important for early adulthood. It is well-established that the optimal attainment of PBM can delay the onset of secondary osteoporosis later in life [1]. Due to the limited window of opportunity to build healthy and strong bones, it becomes imperative to examine lifestyle factors in young adults, which could affect the attainment of their PBM.

According to the 2014 national survey on Drug Use and Health [7], almost 60% of college-aged students (18–22 years) drank alcohol in the past month and almost two out of three of them were considered binge drinkers during that same timeframe. This is important because alcohol can affect skeletal health in multiple ways. Conflicting relationships between alcohol consumption and bone health have been reported relative to sex [8], age [9,10], and drinking patterns [11]. For example, heavy alcohol consumption is associated with a reduced bone mass and increased fracture risk [11], whereas moderate alcohol use is associated with a higher bone density in some studies [12,13,14], especially in women [9]. The conflicting results of alcohol intake on bone may be explained, in part, by its complex relationship with gonadal hormones [10], altered endocrinal signaling [10,15], and the association of alcohol intake behavior with other lifestyle choices [10]. All of these factors can interplay to produce conflicting results on human skeletons. Unfortunately, most of the previous studies investigating the relationship between alcohol consumption and areal bone mineral density (aBMD) have focused on either middle-aged/older men and/or middle-aged/postmenopausal women [16,17,18,19,20]. Very few studies have examined the relationship between alcohol and bone in young adults [21,22,23].

Bone-loading physical activities play a critical role in bone accrual during early adulthood compared to middle adulthood ages and could thus affect the attainment of PBM [24]. Previous findings have indicated that bone-specific loading exercise is a significant factor increasing site-specific bone mass before the PBM is reached, and consistently positive correlations have been reported between high-intensity weight-bearing training interventions and aBMD, and bone strength [25,26]. High-impact or high-intensity weight-bearing activities can produce an osteogenic effect via increased bone turnover [27], decreased osteocyte apoptosis, and an improved viability of osteocytes [28]. Recently, the bone-specific physical activity (BPAQ) score has been widely used to estimate its relationship with aBMD (g/cm^2^) and volumetric bone mineral density (BMD) (vBMD, g/cm^3^) in various populations [3,25,29,30]. However, limited data exist on identifying the “threshold” of physical activity needed to improve and/or maintain bone health in college-aged women [31]. Notably, college-aged populations likely overestimate their body images [32] and may have a propensity to adopt unhealthy diet patterns negatively affecting their PBM. It has been shown that the body mass index (BMI) was positively associated with aBMD in pre- and postmenopausal women [33,34]. Interestingly, BMI is ethnicity-dependent and can differentially affect various physiological measures [35], including aBMD [36]. This becomes important because very little is known about the relationship of ethnic-specific BMI with aBMD in young populations. Knowledge of these relationships would provide us with insight into various factors that may affect aBMD in young women.

Therefore, the purpose of this cross-sectional study was to investigate the differences in aBMD of the non-dominant hip and lumbar spine in healthy college-aged female students based on alcohol consumption (frequency and drinking amount), bone-loading physical activity history (tertile in lowest, mid, and highest), and BMI (Asia-Pacific BMI). We hypothesized that there would be group differences in aBMD variables based on the frequency and drinking amount per occasion. Moreover, we hypothesized that there would be positive relationships among aBMD, the highest BPAQ score, and normal/overweight in Asia-Pacific BMI in young women.

## 2. Materials and Methods

### 2.1. Design and Participants

This study was approved by the Korean Ministry of Health and Welfare Research Ethics Committee (No. 2015-0016-001). All participants provided written informed consent before the data collection.

A cross-sectional design was used in the present study. We recruited participants from March to July in 2016. One hundred and twelve college-aged female students between the ages of 19 and 26 years were recruited from the universities in Seoul and Gyeonggi province, South Korea, via flyers posted on the university websites and mailed to prospective participants. Participants were included if they had regular menstrual cycles and were free of bone disease and not taking any medications, such as corticosteroids, known to affect bone density. Volunteers who were smokers (*n* = 13), pregnant, or taking hormonal birth control medication (*n* = 25) and exceeded the body mass limit of the dual energy X-ray absorptiometry (DXA, 136 kg) were excluded from our study. All participants visited the Seoul Sok Medical Center to complete their measurements. The Korean Ministry of Health and Welfare Research Ethics Committee approved all procedures. Written informed consent was obtained from all participants before testing.

### 2.2. Anthropometry and Body Composition

We measured the standing height (cm), body mass (kg), Body Mass Index (BMI, kg/m^2^), fat-free mass (FFM, kg), and % body fat using X-scan plus II (Hospital body Composition Analyzer, Jawon Medical, Gyeongsan, South Korea). Participants were encouraged not to drink alcoholic beverages for 48 h before the test or participate in any vigorous exercise 12 h before the test so that they were in a euhydrated state. They were also asked not to have any meals, including caffeinated beverages, 4 h before the test. After changing into scrubs, participants removed all pieces of jewelry and socks and then stood on the X-scan plus II equipment. A single qualified technician conducted all measurements, following the manufacturer’s protocol. Based on the Asia-Pacific BMI, participants were classified in four groups: (a) underweight (BMI < 18.5), (b) normal (BMI = 18.5–22.9), (c) overweight (BMI = 23–24.9), and (d) obese (BMI ≥ 25) [37].

### 2.3. Assessment of Alcohol Consumption

The alcohol consumption was determined by the frequency and amount of alcohol intake during the past 12 months using self-reported questionnaires. The alcohol consumption questionnaire had two sections. In the first section, the frequency of drinking was divided into four groups: never (non-drinker), less than once per month, 2–4 times per month, and ≥2 times per week. In the second section, participants were asked to choose the drinking amount per occasion. We followed the definition of one “standard” drink in the UK, which contains 8 g of pure alcohol [38]. The items of the drinking amount per occasion included 220 mL of regular beer (4% of alcohol), 50 mL of Korean distilled liquor (Soju) (20% of alcohol), 240 mL of Makgeolli (Korean traditional rice wine) (7% of alcohol), and 90 mL of wine (12 % of alcohol) [39].

### 2.4. Areal Bone Mineral Density

We used dual energy X-ray absorptiometry (DXA, GE Lunar Prodigy, enCORETM 2002 Software, Madison, WI, USA) to assess the aBMD of the lumbar spine (L1–L4) and the non-dominant side of the proximal femur (total hip, TH; femoral neck, FN; femoral trochanter, FT). A menstrual history questionnaire was used to determine if the participant was pregnant. We provided the scrubs for all participants and participants removed all metal or plastic objects before DXA testing. The participants were asked to lie down on the DXA table in the supine position. For the lumbar spine measurement, a block-shaped cushion was placed under the participant’s feet to flatten out the small of the back and open disk spaces for the highest quality and most accurate lumbar spine images. For the non-dominant side of the proximal femur, the participant’s feet were secured by the DualFemurTM (GE Healthcare, Chicago, IL, USA) positioner to maintain the appropriate internal rotation of the femur. Quality assurance scans were performed daily and qualified technicians, who followed the standard operating procedures of our study, conducted all measures during the study period.

### 2.5. Bone-Loading Physical Activity

A single qualified researcher administered and analyzed all the BPAQ questionnaires using an online BPAQ calculator (www.fithdysign.com/BPAQ/) [40]. Algorithms used to analyze BPAQ responses have been described elsewhere in detail [29]. After sufficient explanation, participants were asked to fill out two independent sections of the BPAQ questionnaire: the past (one year of age to 12 months before testing, pBPAQ) and current (the past 12 months, cBPAQ) periods of historical physical activity related to bone loading. The total period (total BPAQ) was calculated as the average of pBPAQ and cBPAQ scores.

### 2.6. Calcium Intake

A 24-h dietary recall was used to estimate the daily calcium intake. Participants were asked to complete a questionnaire, recalling food intake for the past 24 h, which included listing the following: (1) food/drink items with brand names, (2) the amount ingested, (3) method of preparation with as much detail as possible, and (4) the dosage and frequency of current calcium supplements taken by the participants. A single qualified tester then used the information provided in this questionnaire to analyze the daily calcium intake using the Computer-Aided Nutritional analysis program (CAN-Pro 4.0, The Korean Nutrition Society, Seoul, South Korea).

### 2.7. Statistical Analyses

We performed all analyses using SPSS for Mac version 25 (SPSS, Inc., Chicago, IL, USA) and data are reported as the mean ± standard error of the mean (SEM). A one-way analysis of covariance (ANCOVA) was conducted to compare the aBMD between categories of alcohol consumption, bone-loading physical activity, and BMI, adjusting for relevant covariates such as menarche age, fat-free mass, and calcium intake. Levene’s test and normality checks were carried out and the assumptions met. If the main ANOVA was significant, Bonferroni post hoc tests were carried out to determine site-specific differences in the aBMD of the lumbar spine and the non-dominant side of the proximal femur among the groups. We set the level of significance at *p* < 0.05.

## 3. Results

Table 1 displays the physical characteristics, body composition, daily calcium intake, total BPAQ score, and aBMD variables of our study participants.

As shown in Table 2, there were significant group differences in the aBMD of the total hip (*p* = 0.002), femoral neck (*p* = 0.049), and femoral trochanter (*p* = 0.008), based on the frequency of drinking alcohol after adjusting for age, menarche age, BMI, FFM, calcium, and total BPAQ. Post hoc tests showed ≥2 times per week was associated with greater aBMD of the TH (*p* = 0.04–0.002) and FN (*p* = 0.043) compared to other groups and 2–4 times per month, respectively. Moreover, greater aBMD of the FT was found in those drinking ≥2 times per week compared to those drinking less than once and 2–4 times alcohol per month (*p* = 0.007). We did not find any significant group differences in the aBMD of the lumbar spine (*p* > 0.05). No significant differences were found between the drinking amount per occasion and aBMD variables in any of the groups (*p* > 0.05).

**Figure 1:**
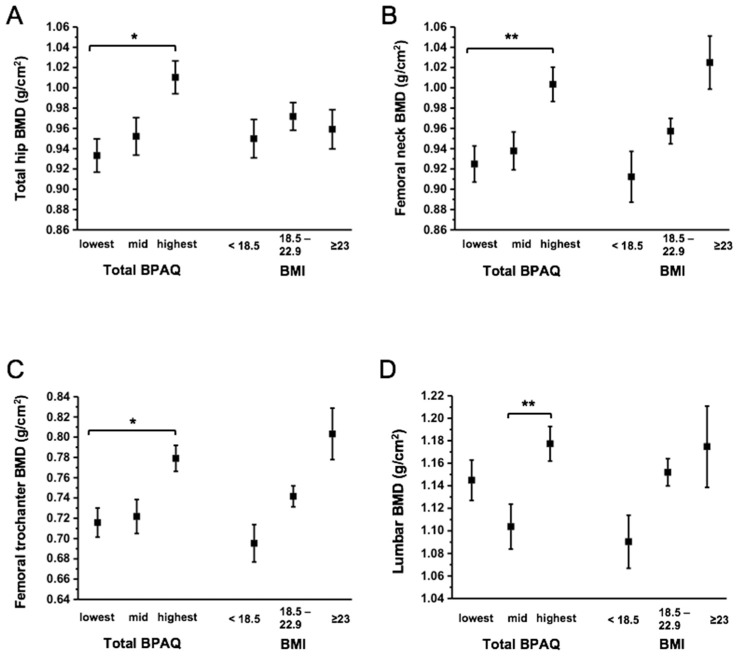
Differences in aBMD for the (**A**) total hip, (**B**) femoral neck, (**C**) femoral trochanter, and (**D**) lumbar spine based on the classification of the bone-loading Physical Activity Score (BPAQ) and Body Mass Index (BMI). Total BPAQ covariates: age, BMI, fat-free mass, menarche age, and calcium intake; BMI covariates: age, total BPAQ, fat-free mass, menarche age, and calcium intake. * *p* < 0.05, ** *p* < 0.001.

In our study, the total BPAQ was used to divide our participants into three groups (lowest, mid, and highest). We found that there were significant differences between total BPAQ score and aBMD variables among groups, after adjusting for age, menarche age, calcium, BMI, and FFM (*p* = 0.002–0.003). As shown in Figure 1, post hoc tests showed that the highest group of total BPAQ had greater aBMD of the TH, FN, and FT compared to the lowest (*p* = 0.023–0.009) and mid total BPAQ groups (*p* = 0.004–0.009). Additionally, the highest group had greater aBMD of the lumbar spine compared to the mid BPAQ group (*p* = 0.001). We noted no significant group differences in aBMD variables (*p* > 0.05) based on the classifications of BMI.

## 4. Discussion

In our cross-sectional study, we found that among modifiable lifestyle factors, bone-loading physical activity is a significant factor increasing bone mass before peak bone mass formation, which is normally achieved in the third decade of life. Moderate alcohol intake shows greater aBMD, whereas BMI is not a significant factor in college-aged women.

Similarly, the highest aBMD was exhibited in postmenopausal women (mean 62.8 ages) who drank 2–3 times per week compared to the non-drinkers [19]. In terms of volumetric BMD (vBMD, g/cm^3^) measured by high-resolution peripheral quantitative computed tomography, Paccou et al. [17] found that men (mean 76.1 years) who consumed low amounts of alcohol had a lower vBMD, but women (mean 76.5 years) with moderate/high alcohol intake had a significantly higher vBMD. Our study did not find differences in aBMD of the lumbar spine, but lighter drinkers (1–9 g/day) exhibited higher aBMD of the lumbar spine in elderly women (50 to 79 years) compared to non-drinkers [16]. The mechanisms underlying alcohol effects on the skeleton are very complicated and remain obscure. However, there is evidence that a moderate alcohol intake could have positive effects on the bone density, but exceeding this level shows negative effects on the skeleton [14]. Garddini et al. [10] defined moderate drinking as the regular (≥3 d/week) consumption of ≤14 g/d EtOH for women and ≤28 g/d EtOH for men, suggesting that light to moderate alcohol consumption may exert beneficial effects on aBMD, but heavy alcohol consumption has negative effects on bone quality. Our results are partially in agreement with previous findings reporting beneficial effects on aBMD (≥2 times per week) [10,14,19]. This could occur, in part, due to decreased bone resorption [41]. Further, dietary silicon, which may be present in a high quantity in alcoholic beverages, can help increase BMD by its anti-resorptive and anabolic effects [42]. Alcoholic beverages also contain various phenolic compounds such as resveratrol, flavonols, anthocyanins, and hydroxycinnamic acids [41]. A review paper showed that phenols can inhibit bone resorption and inflammatory cytokines, and enhance the anabolic effect via increased bone formation [41].

In contrast to our findings, Seo et al. [21]. found that both higher-frequency and Alcohol Use Disorders Identification Test scores (AUDIT scores, 16–17 considered as harmful drinking) had a negative impact on the aBMD of TH and FN, but not on the lumbar spine, in Korean young women (19 to 30 years of age). They suggested that no beneficial relationships were found between moderate drinking habits and bone health. Labrie et al. [22] reported that the frequency of heavy episodic drinking (four or more drinks within a 2-h period on 115 or more occasions) between the freshman year of high school and the sophomore year of college was negatively associated with aBMD of the lumbar spine in young women (18 to 20 years of age). In young men (16 to 18 years of age), those with a moderate intake (12–21 units week^−1^) had the highest aBMD of TH and FN, but the highest alcohol intake group (>21 units week^−1^) had the lowest aBMD [18]. In the current study, we collected the past 12 months of drinking frequency and amount per occasion using self-reported questionnaires. Some discrepancies among the studies could be partly due to different categories (frequency, amount per occasion, units, and AUDIT scores) of alcohol consumption in diverse age groups. Overall, most previous findings did not find a relationship between alcohol consumption and aBMD of the lumbar spine; however, aBMD of the FN was the most sensitive region for predicting its relation to alcohol consumption.

Lifestyle factors during the bone acquisition phase are critically important to the achievement of PBM in young women [1]. Bone-strengthening physical activities (e.g., jumping, running, and high-intensity resistance training) can play an important role in increasing site-specific aBMD during this critical period [25,26]. Previous studies have consistently found positive associations between aBMD/vBMD and bone-loading physical activities estimated by BPAQ scores [3,25,29,30]; however, its threshold to improve bone health, especially in young women before reaching the PBM, has not been well-studied. Our study divided our participants into three groups (highest, mid, and lowest) based on total BPAQ scores, suggesting that the highest group had greater aBMD of the non-dominant hip and lumbar spine compared to the lowest and mid groups. In fact, among modifiable lifestyle factors in college-aged young women, engaging in high-impact physical activities could significantly contribute to increasing the PBM. Consistently, total BPAQ scores were more significant indices for predicting aBMD and bone strength in healthy young women (18.0–30.2 years) compared to middle-aged premenopausal women (35.6–50.9 years) [30]. Increased high-impact physical activity, specifically during the growth period, is beneficial for bone and helps attain optimal PBM [43]. Furthermore, the beneficial effects of past physical activity carry into adulthood [43]. During the growing years, high-impact physical activity can enhance bone accretion, bone structure, and bone density [43]. Even if we speculate that high-impact physical activity tends to be diminished in middle-aged or older women, it is notable that the beneficial effect of high-impact physical activity during growing years can partially translate to late adulthood [43].

Besides, one of our study purposes was to investigate if ethnic-specific criteria for BMI could predict bone health. In the current study, our study participants were all Asians and Asia-Pacific BMI [37] was used to categorize them into three groups (underweight, normal, and overweight and obese). We did not find any differences in aBMD variables in young college-aged women; however, previous studies have shown positive relations to aBMD in pre- and postmenopausal women [33,34]. Since underweight (BMI < 19 kg/m^2^) is one of the risk factors for osteoporosis, maintaining a healthy body weight is also important for overall bone health. Particularly, this young age group tends to exhibit an unhealthy diet pattern which may lead to negative impacts on their bone acquisition before the PBM. The range of BMI in our population was narrow. Therefore, a young age and a narrow range of BMI of our participants may not have allowed sufficient time to exert the measurable effects of BMI on bone in our population.

There are several limitations to our cross-sectional study. Our study used the frequency and amount of alcohol intake during the past 12 months to investigate its relation to bone health using self-reported questionnaires. It is possible that participants’ recall errors and the number of years of drinking affected our results. Additionally, the drinking amount per occasion may not represent their entire drinking events. Since the genetic background significantly affects the metabolism of alcohol, it is difficult to define alcohol-related health effects among populations [41]. Our cross-sectional study design does not determine cause and effect relationships between variables. We only collected the daily calcium intake from the participants, but their overall diet patterns would have influenced alcohol metabolism, as well as bone metabolism.

## 5. Conclusions

Young college-aged women with greater bone-loading physical activity showed greater aBMD at the TH, FN, FT, and lumbar spine, while moderate alcohol intake showed greater aBMD of the TH and FN. However, the amount of drinking alcohol and ethnic-specific criteria of BMI did not affect aBMD variables in college-aged female participants. Among modifiable lifestyle factors, our findings support the importance of bone-loading physical activity in young women. Future studies should include binge drinking patterns and amounts to further explain alcohol and bone growth in young women.

## Figures and Tables

**Table 1:** Descriptive Data for the Study Population (*N* = 112).

Variables	Mean ± SEM	Range
Age (years)	21.9 ± 0.2	19.0–26.8
Height (cm)	161.9 ± 0.5	148.0–174.0
Body Mass (kg)	53.4 ± 0.6	39.0–68.6
Body mass index (kg/m^2^)	20.4 ± 0.2	15.8–26.3
Menarche age (years)	12.5 ± 0.1	9.0–16.0
Fat-free mass (kg)	39.9 ± 0.3	31.5–47.7
% body fat (%)	25.0 ± 0.4	15.2–35.6
Calcium (mg/day)	394.2 ± 21.3	24.7–1070.3
Total BPAQ	16.5 ± 1.0	1.3–49.3
aBMD (g/cm^2^)		
Total hip	0.966 ± 0.010	0.727–1.251
Femoral neck	0.956 ± 0.011	0.693–1.242
Femoral trochanter	0.739 ± 0.009	0.475–1.027
Lumbar spine	1.141 ± 0.011	0.907–1.423

aBMD: areal bone mineral density; BPAQ: bone-specific physical activity questionnaire; SEM: standard error of the mean.

**Table 2:** aBMD according to the frequency of drinking and drinking amount per occasion (means ± SEM).

Classifications	*n* (%)	Total Hip	Femoral Neck	Femoral Trochanter	Lumbar Spine
**Frequency of drink**					
Never	14 (12.5)	0.965 ± 0.018	0.956 ± 0.019	0.747 ± 0.014	1.118 ± 0.035
Less than 1 per month	30 (26.8)	0.947 ± 0.023	0.948 ± 0.024	0.715 ± 0.021	1.143 ± 0.021
2–4 times per month	52 (46.4)	0.951 ± 0.013	0.940 ± 0.014	0.731 ± 0.011	1.125 ± 0.013
≥2 times per week	16 (14.3)	1.052 ± 0.030 ^a^	1.024 ± 0.031 ^b^	0.806 ± 0.025 ^b,c^	1.213 ± 0.030
*p*-value		0.002	0.049	0.008	0.128
**Drinking amount per occasion**					
0–1 glasses	17 (15.2)	0.951 ± 0.018	0.940 ± 0.022	0.735 ± 0.015	1.119 ± 0.029
2–5 glasses	23 (20.5)	0.968 ± 0.025	0.967 ± 0.027	0.735 ± 0.022	1.134 ± 0.020
6–9 glasses	40 (35.7)	0.983 ± 0.018	0.964 ± 0.017	0.751 ± 0.017	1.145 ± 0.018
10 and more glasses	32 (28.6)	0.951 ± 0.019	0.947 ± 0.021	0.731 ± 0.015	1.156 ± 0.020
*p*-value		0.419	0.703		0.944

aBMD: Areal bone mineral density (g/cm^2^); adjusted ANCOVA: age, menarche age, body mass index, fat-free mass, calcium, and total bone specific physical activity score; ^a^ significantly different from all the other groups; ^b^ significantly different from 2–4 times per month; ^c^ significantly different from less than 1 per month.
